# Sensitive detection of specific cell-free DNA in serum samples from sheep with cystic echinococcosis

**DOI:** 10.1371/journal.pntd.0011715

**Published:** 2023-10-19

**Authors:** Mahboubeh Hadipour, Hossein Yousofi Darani, Hamid Talebzadeh, Mohammad Eslamian, Shima Aboutalebian, Majid Fasihi Harandi, Hossein Mirhendi

**Affiliations:** 1 Department of Parasitology and Mycology, School of Medicine, Isfahan University of Medical Sciences, Isfahan, Iran; 2 Department of Surgery, School of Medicine, Isfahan University of Medical Sciences, Isfahan, Iran; 3 Research Center for Hydatid Disease in Iran; Dept. of Parasitology, School of Medicine, Kerman University of Medical Sciences, Kerman, Iran; Wadsworth Center, UNITED STATES

## Abstract

**Background:**

Developing more sensitive methods for the diagnosis of echinococcosis is essential. In this study PCR assay for sensitive detection of specific cell-free DNA (cfDNA) of *Echinococcus granulosus sensu lato* in the sera of the sheep naturally infected with echinococcosis was investigated.

**Methods:**

To extract cfDNA from 35 infected sheep, the modified phenol-chloroform method was used for two different volumes (0.5 and 2 ml) of serum samples. From each extracted sample, two DNA volumes (5 and 10 μl) were amplified using both standard PCR and semi-nested PCR targeting NADH dehydrogenase subunit I.

**Results:**

Standard and semi-nested PCR on 0.5 ml of serum samples detected *Echinococcus* DNA in 8 and 12 out of 35 sheep, respectively; however, using 2 ml of serum samples, they detected 24 and 27 samples. By increasing the volume of template DNA, the PCRs could detect 29 and 33 out of 35 samples. The results were confirmed by sequencing of randomly selected PCR amplicons and comparing them with GenBank databases.

**Conclusions:**

Larger volumes of serum for DNA extraction, greater volumes of DNA template for PCR, and employing a semi-nested PCR protocol, increased the sensitivity of PCR to 95%. This approach can also be applied to the diagnosis of echinococcosis in humans.

## Author summary

Cystic echinococcosis (CE) is a disease caused by the larvae of the worm *Echinococcus granulosus*. Ingesting the eggs of these worms which are excreted in the feces of infected dogs, can result in the formation of cysts in the liver or lungs of humans or herbivorous animals. Although ultrasound is commonly employed for diagnosing these cysts, hydatid cysts can frequently be misdiagnosed as cystic lesions of different origins. Hence, the necessity for faster, more accurate, and non-invasive diagnostic methods is evident. In this study, serum samples were collected from 35 sheep with hydatid cysts, and DNA was extracted and purified. Specific primers were then utilized to amplify the free DNA of the sera via polymerase chain reaction (PCR). Our findings indicate that the diagnostic sensitivity of this disease can be enhanced up to 95% by increasing the volume of serum, employing larger amounts of DNA template in the PCR reaction, and utilizing nested PCR assay. If applied to human samples, this method holds great promise for significantly improving the sensitivity and accuracy of CE diagnosis.

## Introduction

Cystic echinococcosis (CE) is a neglected tropical zoonosis caused by larvae of the tapeworms *Echinococcus granulosus sensu lato*. In the life cycle of the parasite, canines are known as the definitive hosts in which the adult tapeworms develop in the small intestine after ingesting livestock offal infected by hydatid cysts. The dogs excrete the infective eggs into the environment through defecation. The eggs are ingested by livestock as intermediate hosts, followed by developing the eggs into the larvae known as hydatid cysts which are located in different organs, especially in the liver and lungs [1,2]. Human indeed is an accidental host that is not directly involved in the parasite life cycle.

Cystic echinococcosis is a cosmopolitan disease with an annual incidence ranging from <1 to 200 per 100,000 in humans and a mortality rate of 2% which may be increased if sufficient care management is not provided [3]. Every year, CE causes the loss of about 1 million disability-adjusted life years (DALYs) and USD 3 billion in expenses, including treatment and livestock losses [4]. Elimination or control of CE is difficult, thus active surveillance of the disease should be considered a realistic goal by detecting the parasite in livestock particularly in sheep herds [5]. Carcass inspection is the primary method for identifying sheep with cystic echinococcosis, which has a great limitation. Serological diagnosis of infection in animals has received less attention despite the potential value of such tests for hydatid control programs. The tests have limitations including low specificity (cross-reaction with other taeniid cestode species) and the complexity and unstablity of natural antigen sources [6].

Diagnosing hydatid cysts in humans is based on clinical findings, imaging, and immunodiagnostic tests [7,8]. Clinical manifestations of the disease are not specific and often appear in the later stages of the disease [9,10]. Ultrasound (Us) imaging is considered a safe, low-cost, and rapid technique for diagnosing, following up, and screening of CE [11]. However, hydatid cysts can frequently be misdiagnosed as cystic lesions of different origins [12,13]. Serologic tests based on detecting a parasite antigen or host antibody can be helpful [14,15]; however, they have limitations including cross-reactions, variation in sensitivity and specificity in different conditions, and a lack of differentiating the present and past infections [16–18].

DNA-based assays such as conventional polymerase chain reaction (PCR), real-time PCR, and loop-mediated isothermal amplification (LAMP) have been used for accurate assessment of *E*. *granulosus* prevalence in definitive host feces [19–22] or identification of different genotypes in intermediate host [23,24].

Cell-free DNAs (cfDNAs) are extracellular nucleic acid fragments that can be released into body fluids from any exotic cell or organ in various tissue due to apoptosis, cell necrosis, and secretions. cfDNAs can be detected in body fluids including whole blood, serum, plasma, urine, and saliva. The typical features of cfDNAs are small length, low richness, and fast degradation. It is estimated that the size of cfDNA varies from ~40–200 base pairs (bp), with a dominant peak of 166 bp. The concentration of cfDNA in 1 mL of human plasma in normal conditions is estimated to be around 1~10 ng; however, under certain circumstances (more cell death and/or deficient removal of the dead cells) or after exercise, the concentration increases to hundreds of nanograms [25]. They have a half-life of about 10–15 min and are typically removed by the liver. Recently, several studies have demonstrated that specific circulating cfDNAs are promising diagnostic targets for some human diseases, such as autoimmune diseases, metabolic disorders, and cancers [26–28]. Given the current limitations in the laboratory diagnosis of echinococcosis by immunological and imaging approaches, new diagnostic approaches based on the detection of parasite cfDNA should be considered. Recent studies have reported the presence of *Echinococcus*-derived cfDNA in plasma, serum, and urine samples of infected patients, offering a promising approach toward non-invasive diagnosis. However, PCR or qPCR methods for detecting *Echinococcus* cfDNA in patients’ sera have shown limited sensitivity, with reported rates of 20–25%. Thus, these methods have yet to be improved and established as reliable diagnostic tools for echinococcosis [25]. To address this issue, our study aimed to enhance molecular techniques’ sensitivity, specificity, and diagnostic validity for detecting *E*. *granulosus sensu lato* cfDNA. In the present study we present a semi-nested PCR assay for detecting cfDNA in sheep sera with a potential application for diagnosing CE in human samples. We hope that this study opens up a new avenue for the diagnosis of human echinococcosis in the future.

## Materials and methods

### Ethics statement

This project involvied only animal (sheep) and approved by the ethics committee of Isfahan University of Medical Sciences, Isfahan, Iran (IR.MUI.MED.REC.1400.101).

### Primer designing and specificity testing

*E*. *granulosus sensu lato* DNA sequences *(*NC_044548.1, MG672262.1, MG672261.1, and MG672286.1) retrieved from the deposited data in GenBank, were aligned and evaluated using Geneious Prime software (https://www.geneious.com). After careful analysis of the sequences, two mitochondrial genes, i.e. NADH dehydrogenase subunit I (nad1) and Cytochrome C oxidase subunit I (cox1), were considered the optimal targets for PCR amplification, and four sets of primer pairs were designed for PCR and semi-nested PCR. Primers were synthesized by Metabion company (Germany). Since the preliminary tests for setting up the PCR showed a higher sensitivity of nad1 than cox1, the former was chosen as the target for the subsequent experiments. To evaluate the specificity, PCR was carried out for each set of primers using DNAs extracted from *E*. *granulosus* protoscolex and hydatid cyst fluid as the positive controls, and a panel of non-target *E*. *granulosus* DNAs (Table 1) as the negative controls. Features of the primers and the negative controls are presented in Table 1.

**Table 1:** The primers and controls used for the PCR detection of cfDNA in the sera collected from 35 sheep with cystic echinococcosis.

Targeted organism	Primers	Targeted region	Product size	Positive controls	Negative controls
*E*. *granulosus*	Echino.nad.F1:TATCGGTATGTTGGTGTTAGTG	NADH dehydrogenase subunit I	F1, R1: 245 bp	Protoscolex, Hydatid cyst fluid	***Fasciola hepatica*,** ***Taenia saginata*,** ***Toxocara canis*,** ***Toxocara catti*,** ***Enterobius vermicularis*,** ***Hymenolepis nana*,** ***Trichuris trichiura*,** ***Dicrocoelium dendriticum*,** ***Giardia duodenalis***
	Echino.nad.R1: AGTAAGCCACATCGACCATAA				
	Echino.nad.R2: CAGACTCATGCAACACAAAA		F1, R2: 172 bp		
	Echino.cox.F1: TAGTGCTAATTTTGATGCGTTTGG	Cytochrome c oxidase subunit I	F1, R1: 254 bp		
	Echino.cox.R1: CACTATAAAAGAAACAACCCATCA				
	Echino.cox.F2: TGGGTTGTTGTTTGCTATGTTT		F2, R2: 158 bp		
	Echino.cox.R2:ACCAAGTAAACACCTTTATACCAGT				

### Sample collection

The study population of this work consisted of the sera collected from 35 sheep naturally infected with hydatid cysts in the liver or lung that were slaughtered in a local abattoir in Isfahan Province, Iran. The size of almost all cysts was over 2 cm. The golden standard for diagnosing CE in sheep was the observation of typical hydatid cyst(s) in the viscera following routine veterinary inspections as well as the confirmations made by the research team. Fifty milliliters of the peripheral whole blood were collected from each sheep, allowed to clot by leaving it undisturbed at room temperature, and transferred to the diagnostic molecular laboratory at the School of Medicine, Isfahan University of Medical Sciences. The serum of each sheep was separated into a new tube and stored at -20°C until use. Twenty normal human sera taken from individuals who had reffered to the clinical diagnostic laboratory for routine check up, were used as the negative control samples. Since some asymptomatic individuals may have low stage echinococcosis, and to ensure that individuals whose sera are used as negative control samples are truly negative, 10 serum samples were randomly selected and tested by a commercial echinococcosis ELISA kit (Pishtazteb ELISA kit, Iran).

### DNA extraction

The total DNA was extracted from two different serum volumes (0.5 and 2ml) by phenol-chloroform-isoamyl alcohol method with slight modification. Each sample was mixed with lysis buffer (100 mM Tris pH 8, 10 mM EDTA, 100 mM NaCl, 1% SDS, 1% Triton X-100) and saturated phenol (pH 8), shaken for a few min, and centrifuged at 5000 rpm for 5 min. The supernatant was mixed with an equal volume of phenol-chloroform-isoamyl alcohol (25: 24: 1), shaken for a few min, and centrifuged at 5000 rpm for 5 min; the supernatant was mixed with an equal volume of chloroform-isoamyl alcohol (24: 1) and centrifuged for 5 min at 5000 rpm, the supernatant was mixed with an equal volume of isopropanol and 0.1× volume of Sodium acetate (3 M, pH 5.2) and incubated overnight at -20°C, then was centrifuged at 12,000 rpm for 10 min, 500 μL 70% ethanol was added to the sediment and centrifuged at 12,000 rpm for 10 min, and the pellet was resolved in distilled water as the template DNA for PCR. The materials used for DNA extraction for both serum volumes (0.5 and 2ml) are listed in Table 2.

**Table 2:** Materials used in DNA extraction from 0.5 and 2ml serum samples.

Materials	0.5 ml	2ml
**Lysis buffer concentration (the volume)**	1*×* (400 μl)	5*×* (200 μl)
**Phenol**	300 μl	1000 μl
**Water used for DNA elution, (the amount used for PCR)**	30 μl (5 or 10 μl)	60 μl (10 μl)

### Standard and semi-nested PCRs

**Fig 1:**
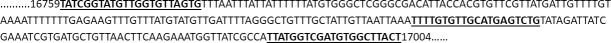
**The location of the amplified gene fragments and the primers (nad F1, nad R2, and nad R1) in the PCR and nested PCR on “*Echinococcus granulosus* mitochondrion, complete genome, NCBI Reference Sequence: NC_044548.1”.** The numbers indicate the beginning and the end of the fragment in the genome.

Both standard and semi-nested PCR amplifications were used to detect *E*. *granulosus-*specific cfDNA of the mitochondrial gene nad1. The location of the primers and the gene fragment amplified in the standard and semi-nested PCR are shown in Fig 1. At the beginning of the work, different concentrations (0.3 to 0.7 μM) and the annealing temperatures (55°C to 60°C) of each primer were tested, and the PCR amplification conditions for standard and semi-nested PCR were optimized. For standard PCR, a mix of 5 or 10 μl of DNA extracted from serum samples, 0.4 μM of each primer, 12.5 μl of 2X master mix (Ampliqon, Denmark), and adequate water up to a 25 μl PCR reaction mixture was prepared and amplification was conducted under the following thermal conditions: 95°C 5 min as initial denaturation, 35 cycles of 94°C 20 s, 60°C 1 min, and 72°C 45 s; and a final extension at 72°C for 5 min. For first round of semi-nested PCR, a mixture of 5 or 10 μl of the extracted DNA, 0.4 μM of each primer, 12.5 μl of 2X master mix, and adequate water up to 25 μL were subjected to PCR with the following conditions: 95°C 5 min as initial denaturation, 35 cycles of 94°C 20 s, 59°C 45s, 72°C 30s; and a final extension at 72°C for 2 min. In the second PCR round, 2 μl of 1/50 diluted PCR product, 0.3 μM of each primer, 12.5 μl of 2X master mix, and enough water up to 25 μL were used. The PCR was then conducted under the conditions of 95°C for 2 min; 30 cycles of 94°C for 15 s, 60°C for 45 s, 72°C for 20 s; and a final extension at 72°C for 2 min.

The PCR products and a 100 bp DNA size marker (SMOBIO 100 bp ladder, Taiwan) were electrophoresed on 1.5% agarose gel containing 5 microgram/ml ethidium bromide in TBE buffer (90 Mm Tris, 90 Mm Boric acid, 2 Mm EDTA) and visualized by gel documentation.

### Sequencing

To confirm the specificity of the semi-nested PCR, four positive PCR products were randomly selected and purified manually by ethanol precipitation and subjected to sanger sequencing (Research Core Facilities Laboratory, Isfahan University of Medical Sciences, Iran) using the forward primer Echino.nad.F1, and the sequences were BLAST-analyzed (https://blast.ncbi.nlm.nih.gov/Blast.cgi).

## Results

Using positive and negative controls, the initial evaluation of the designed primer sets in standard and semi-nested PCRs confirmed the specificity of the primers. In agarose gel electrophoresis of the PCR products from nad1 and cox1 targets of *E*. *granulosus*, the primers generated amplicons with expected sizes of 172 bp and 158 bp, respectively. The results revealed that a concentration range of 0.3 to 0.4 μM for the primers provide optimal amplification results and using different annealing temperatures ensured the presence of the expected PCR products. With increasing the amount of DNA, the PCR sensitivity was increased (Tables 3 and 4), however, the assessment of *E*. *granulosus* cfDNA detection of cox1 was discontinued following preliminary low-sensitivity results (Table 3).

**Table 3:** Detection of cfDNA using cox1 primer from different volumes of sheep serum using standard and semi nested PCRs.

	The volume of serum sample	
**PCR protocol**	500 μl (10 μl DNA template)	2000 μl (10 μl DNA template)
**Standard PCR (n = 35)**	5 (14.3%)	23 (65.7%)
**Nested-PCR (n = 35)**	8 (22.9%)	27 (77.1%)

**Table 4:** Detection of cfDNA in different volumes of sheep sera using standard and semi nested PCRs and nad1 primer.

	**The volume of serum sample**			
Detection Methods	500 μl		2000 μl	
	**The volume of used template DNA**			
	10 μl	5 μl		10 μl
Standard PCR (n = 35)	8 (22.9%)	24 (68.6%)		29 (82.7%)
Nested-PCR (n = 35)	12 (34.3%)	27 (77.1%)		33 (94.3%)

**Fig 2:**
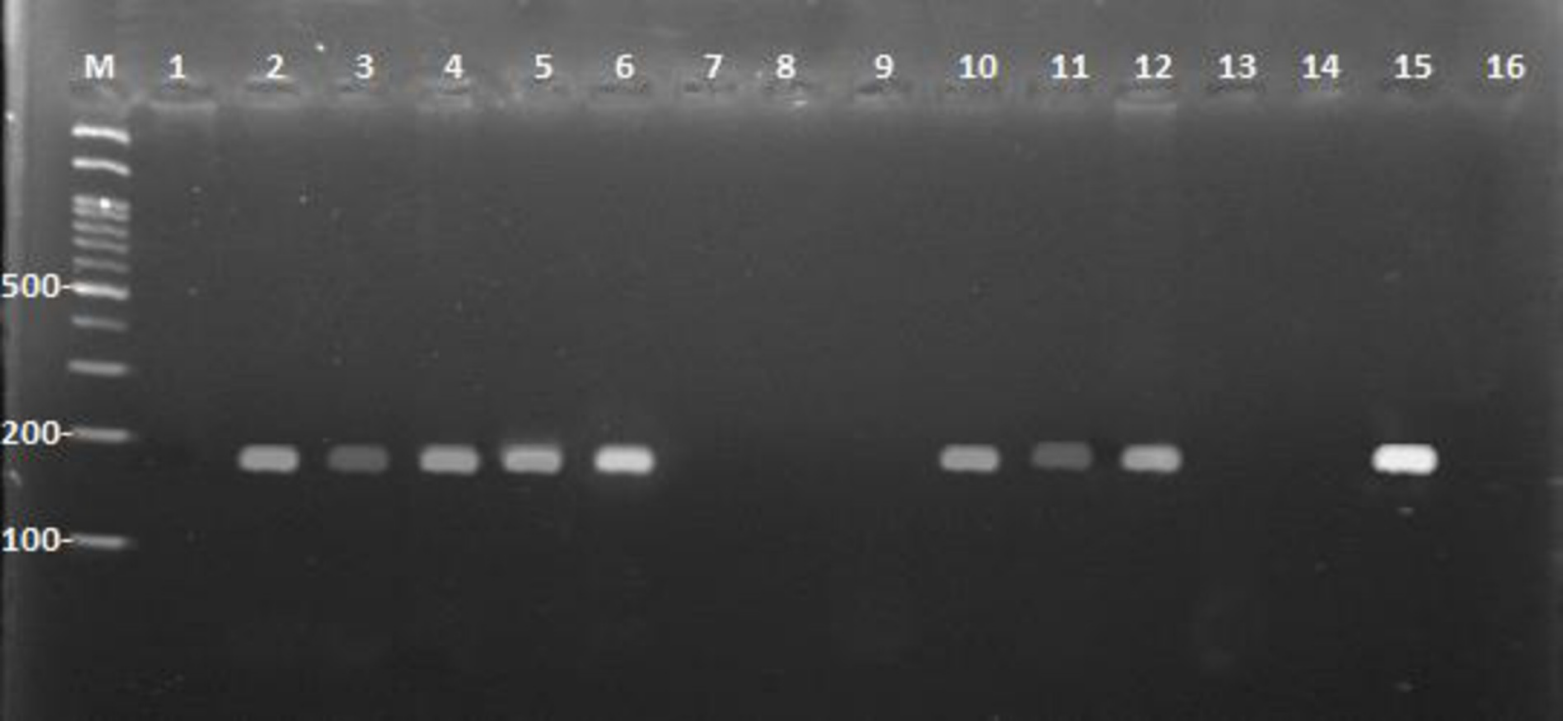
**Agarose gel electrophoresis of the standard PCR and semi-nested PCR products from sheep serum samples in 500 and 2000 ml volums.** Lane M. 100 bp DNA molecular size marker, lanes 1 and 7. standard PCR in the 0.5 ml volume, lanes 2 and 8. semi-nested PCR in the 0.5 ml volume, lanes 3 and 9 are standard PCR in the 2 ml volume and the 5 μl template DNA, lanes 4 and 10 are semi-nested PCR in the 2 ml volume and the 5 μl template DNA, lane 5 and 11 are standard PCR in the 2 ml volume and the 10 μl template DNA, lanes 6 and 12 are semi-nested PCR in the 2 ml volume and the 10 μl template DNA, lane13 is a representative negative clinical samples, lanes 14 and 15. negative and positive controls, respectively.

To maximize the detection of *E*. *granulosus* nad1 cfDNA in serum samples of the sheep naturally infected with hydatid cyst, two formats of PCR were run, different volumes of serum samples for DNA extraction were used, and different amounts of template DNA were applied in the PCR reaction. Application of a 10 μl aliquot of the DNA extracted from 0.5 ml sera in standard PCR yielded a band of around 170 bp in 22.9% (8/35) of the sera, while in semi-nested PCR, 34.3% (12/35) sera were positive (Table 4). On the other hand, with 5 μl DNA extracted from 2 ml serum samples in standard PCR, a 170 bp band was seen in 68.6% (24/35) sera, while in semi-nested PCR, 77.1% (27/35) sera were positive. In standard PCR using a 10 μl aliquot of DNA extracted from the 2ml samples, a single 170 bp band was observed in 82.7% (29/35) sera; however, semi-nested PCR could produce this amplicon in 94.3% (33/35) sera (Table 4). No bands were seen in PCR amplification of the reactions with negative controls (water instead of DNA), all 20 normal human sera, and all organisms used as the negative samples for evaluating the specificity. Additionsally, the commercial ELISA test yielded negative results for all normal human sera samples, indicating the absence of any detected antibodies, while the positive control of the kit successfully produced a positive result. Therefore, considering the observation of cysts in the sheep viscera as the gold standard, the highest sensitivity and specificity of the molecular methods used in our study were estimated as 94.29% and 100%, respectively (Table 4). Fig 2 shows the agarose gel electrophoresis of amplified DNAs from two representative sheep serum samples, used in two serum sample volumes and two different amounts of template DNA used in standard and semi-nested PCR.

Comparison of the sequences obtained from four randomly selected PCR amplicons with GenBank databases confirmed that all sequences are belong to the mitochondrial nad1 region of *E*. *granulosus* (apparently the genotype G1) having significant identity with reliable sequences already deposited in GenBank.

## Discussion

Cystic echinococcosis has been registered by the World Health Organization as one of the 20 neglected diseases targeted for control or elimination until 2030. Limitations in diagnostic methods, the low efficacy and side effects of the available drugs, and the problems in the implementation of preventive measures are among the challenges of CE management and control [29]. There are several approaches for CE control and prevention, including an effective livestock vaccine, dog deworming, tailored educational programs, more effective antiparasitic treatments, and the development of better diagnostics for humans as well as the definitive and intermediate hosts humans [5].

Sheep as a domestic herbivorous animals is the key intermediate host of *E*. *granulosus* in most endemic regions of the world. The disease causes much economic damage due to increased mortality, decreased productivity, loss of body weight, and high costs of sanitary, highlighting the need for a sensitive and accurate diagnosis technique [30].

The most common method for diagnosis of cystic echinococcosis in domestic livestock is the post-mortem examining of the organs and tissuses at abattoirs. Imaging has been used effectively on a small scale but is not suitable for large-scale epidemiological surveys. The potential of ultrasound investigation for CE diagnosis has shown promising sensitivity (>70%), however, there is variability in specificity caused by the common ovine cysticercosis caused by *Taenia hydatigena* in many endemic regions [31]. Serological tests for CE in sheep have been generally inconsistent and contradictory, attributed to main challenges in serological diagnosis such as cross-reactivity with other cestodes, false positive results and low sensitivity due to limited immune responses to parasite antigens [32].

cfDNA has been largely applied for detecting parasites such as *Plasmodium*, *Trypanosoma*, *Leishmania*, *Schistosoma*, and *Wuchereria* spp. with high accuracy [33]. The limited studies have detected *Echinococcus* cfDNA in human patients, using PCR-based methods, next-generation sequencing (NGS), and DNA-deep sequencing [25]. NGS and DNA-deep sequencing has demonstrated high sensitivity, but due to costs and inaccessibility in most endemic areas, they are not appropriate for routine clinical examination. PCR-based methods had a sensitivity of 20 to 25% for detecting *Echinococcus* cfDNA in human patients’ sera. This low sensitivity of the assays might be due to the low concentration of *Echinococcus* cfDNA in serum and the highly fragmented nature of cfDNA [34]. Moradi et al. reported that the low sensitivity of their PCR method in the detection of *Echinococcus* cfDNA might be because of the primers detecting long cfDNA fragments (400 or 450 bp); therefore, amplification of the shorter fragment was recommended [35]. Baraquin and colleagues detected parasite-derived cfDNA in the serum of humans infected with alveolar echinococcosis (AE). They also detected parasite-derived cfDNA in sera of experimentally infected animals using real time PCR and droplet digital PCR (dPCR). They used two primers selected from two genomic regions, including a nuclear-repeated region (U1 snRNA) and a mitochondrial region (Nad5). All animals infected with AE and only 25% of human samples were positive. They concluded that a very low concentration of cfDNA in samples is the reason for the low sensitivity of their PCR assay [36]. Toribio et al. detected *E*. *granulosus* cfDNA in urine samples of 9 out of 12 patients, by using EgG1Hae III primer to amplify a 133 bp fragment. This primer is present with ~7000 copies arranged in tandem in groups of 2–6 repeats in the *E*. *granulosus* genome and possibly is one of the most highly represented nucleic acid species in the *Echinococcus*-derived cfDNA. In their study, patients that based on imaging methods harbored the active cyst in the liver were positive with the PCR; however, patients harboring calcified liver cysts gave variable results possibly because of the period of calcification and the level of inflammation in the cyst-proximal microenvironment. The sensitivity of the method for identifying the cysts situated outside of the liver, either in inactive or active stages, was less than the sensitivity of the test for liver cysts [37].

In this study, we investigated specific cfDNA detection in the serum samples of sheep infected with *E*. *granulosus* as a diagnostic method. Given the low concentration of cfDNA in serum samples, two different volumes of serum samples and different amounts of extracted DNA were compared. Furthermore, to improve the sensitivity of the detection, standard PCR was compared with semi-nested PCR. After evaluation of the results, we found that a combination of some factors, including the application of a larger volume of clinical specimens, a more amount of template DNA, and a two-step PCR amplification, created a higher level of sensitivity (94.2%) in the detection of a low amount of specific cfDNA. The study proves that parasitic cfDNA can be detected with high sensitivity in intermediate hosts infected with echinococcosis.

Due to the lack of obvious or specific signs/symptoms and the existence of small cysts during the early stages, timely diagnosis of the disease in humans is difficult. Presently, a combination of imaging and serological techniques is recommended for the diagnosis of CE in humans, which is not straightforward and has some limitations. Therefore, developing a sensitive, accurate, and non-invasive diagnosis method is essential. In this study, we demonstrated that *E*. *granulosus* cfDNA present in the serum samples of naturally infected sheep, can be detected with high sensitivity. Hence, it is possible to apply this assay for diagnosing human echinococcosis. Since one of the biggest diagnostic problems is small cysts, thus more investigations are needed using this method on the serum samples of patients with cysts of various stages and sizes.

## Conclusion

We report a sensitivity of 94.2% by an improved PCR method in detecting *E*. *granulosus sensu lato* cfDNA in serum samples of the sheep naturally infected with a hydatid cyst. This high level of sensitivity was related to establishing a new practical protocol based on using a higher volume of sera for DNA extraction, applying a higher amount of DNA in the PCR reaction, and running a semi-nested PCR method. This study provides insights into the diagnosis of human echinococcosis with a higher specificity and sensitivity.
